# Case report: Granulocyte-macrophage colony-stimulating factor sargramostim did not rescue the neutrophil phenotype in two patients with JAGN1-mutant severe congenital neutropenia

**DOI:** 10.3389/fimmu.2024.1373495

**Published:** 2024-09-02

**Authors:** Susan Farmand, Susanne Eva Aydin, Katharina Wustrau, Svea Böhm, Francis Ayuk, Gabriele Escherich, Julia Skokowa, Ingo Müller, Kai Lehmberg

**Affiliations:** ^1^ Division of Pediatric Stem Cell Transplantation and Immunology, University Medical Center Hamburg-Eppendorf, Hamburg, Germany; ^2^ Department of Pediatrics, University Medical Center Ulm, Ulm, Germany; ^3^ Department of Stem Cell Transplantation, University Medical Center Hamburg-Eppendorf, Hamburg, Germany; ^4^ Clinic of Pediatric Hematology and Oncology, University Medical Centre Hamburg-Eppendorf, Hamburg, Germany; ^5^ Department of Hematology, Oncology, Clinical Immunology, University Hospital Tübingen, Tübingen, Germany

**Keywords:** JAGN1 deficiency, severe congenital neutropenia, GM-CSF, SCN6, case report, G-CSF

## Abstract

**Background:**

Homozygous or compound heterozygous mutations in JAGN1 cause severe congenital neutropenia. JAGN1-mutant patients present with severe early-onset bacterial infections and most have been described as low-responders to recombinant granulocyte colony-stimulating factor (G-CSF) therapy. In a murine, hematopoietic JAGN1 knockout model, which displays susceptibility to Candida albicans infection in the absence of neutropenia, treatment with granulocyte-macrophage-CSF (GM-CSF) was able to restore the functional defect of neutrophils.

**Patients:**

We present two unrelated patients with biallelic JAGN1 mutations, who were both treated with subcutaneous GM-CSF (sargramostim) after treatment failure to G-CSF. The first patient was an 18-year-old pregnant woman who received GM-CSF at 12 weeks of gestation up to a dose of 10 µg/kg/d for 7 days. The second patient was a 5-month-old girl who received GM-CSF for a total of 9 days at a dose of up to 20 µg/kg/d. GM-CSF did not increase neutrophil counts in our patients. Treatment was stopped when neutrophil numbers declined further, no beneficial effect was noticed, and patients presented with infections. No adverse effects were observed in either patient and the fetus. Both patients ultimately underwent successful hematopoietic stem cell transplantation.

**Discussion:**

Both patients showed a high recurrence rate of severe infections on G-CSF treatment. GM-CSF therapy did not ameliorate the clinical phenotype, in contrast to the improvement of neutrophil function observed in the JAGN1 mouse model. No major additional extra-hematopoietic manifestations were evident in our patients.

**Conclusion:**

In two unrelated patients, GM-CSF did not have any beneficial effect on neutrophil counts. Patients with JAGN1-mutant SCN with reduced G-CSF responsiveness and elevated infection rate should be evaluated early for stem cell transplantation.

## Highlights

- GM-CSF did not improve neutrophil counts in two unrelated patients with SCN due to JAGN1 mutations.
- Severe infection history and lack of therapeutic benefit by recombinant G-CSF or GM-CSF should prompt consideration of HSCT in JAGN1-mutant SCN.

## Introduction

There are several different monogenetic causes associated with severe congenital neutropenia (SCN), which is usually characterized by peripheral neutrophil counts below 500 cells/µl and a maturation arrest of granulopoiesis in the bone marrow (1–3). In 2014, homozygous mutations in *jagunal homolog 1 (JAGN1)*, coding for a transmembrane protein, were found to be associated with a high risk of early onset bacterial infections due to lack of mature neutrophils (4). The JAGN1 protein is located at the endoplasmatic reticulum, and is essential for differentiation and function of human neutrophils (4). JAGN1-mutant SCN, which is now classified as autosomal-recessive SCN6 (OMIM #616022), is a rare entity with less than 25 cases reported in literature (4–11). Even in study populations with high rates of consanguinity, the documented frequency of JAGN1 is very low (e.g., 1% or 2/216 SCN patients in the Turkish SCN register) (10). In addition to neutropenia, extra-hematopoietic symptoms, such as short stature, skeletal abnormalities, neurodevelopmental delay, and pancreatic insufficiency have been described (11).

Both granulocyte colony-stimulating factor (G-CSF) and granulocyte-macrophage-CSF (GM-CSF) are well-known factors of myeloid cell differentiation. Neutropenia in humans is routinely treated with recombinant human G-CSF (2). While most types of SCN show a rise in neutrophil numbers and an associated reduction of infection episodes with G-CSF treatment (12), most patients with JAGN1-mutant SCN have been described as low responders to this therapy (4). Based on data from a conditional, hematopoietic JAGN1 knockout mouse, Wirnsberger et al. suggested that treatment with granulocyte-macrophage colony-stimulating factor (GM-CSF) might be a potential therapeutic option for JAGN1-mutant patients (13). While the JAGN1 knockout mouse did not show neutropenia, functional neutrophil defects were observed. Treatment with GM-CSF restored the previously defective cytotoxic activity, MPO-release and ability to destroy *Candida albicans* in the murine JAGN1-deficent neutrophils and improved survival during systemic Candida albicans challenge. Furthermore, GM-CSF restored defective fungicidal functions in bone marrow cells of JAGN1-mutant patients *in vitro* (13).

To the best of our knowledge, no GM-CSF-treated JAGN1-mutant SCN patients have been reported in the literature so far. During an early trial reported in 1990 with i.v. GM-CSF including five patients with SCN of unknown genetic origin, only one patient showed an increase in neutrophils, while eosinophils became elevated in the others. Still some clinical benefit (resolution of gingivostomatitis in 2 patients) was noted (12). GM-CSF treatment (3-30 µg/kg/d) was well tolerated over a 42-day period. Another study published in 1989 reported on the beneficial rise in neutrophil counts within 2 weeks of GM-CSF treatment in four patients with severe neutropenia of unknown origin (14). While G-CSF is the standard of care in SCN and effective in more than 90% of patients (15), GM-CSF therapy has more recently been advocated in SCN cases with infections and refractory G-CSF response due to G-CSF receptor deficiency (16, 17). Here, GM-CSF doses as low as 3 µg/kg/d once a week were effective. In these patients, biallelic inherited mutations in the extracellular domain of the G-CSF receptor (CSF3R) had resulted in defective G-CSF signaling (18). In contrast, acquired truncating mutations in the cytoplasmatic region of the G-CSF receptor do not lead to neutropenia, but rather present a risk factor for the development of leukemia in patients with SCN, most likely due to hyper-responsiveness of selected clones to G-CSF (19, 20). Notably, it has been demonstrated that both the risk of developing leukemia and the risk of developing serious infectious complications increases in patients with SCN requiring more than 8 µg/kg/d G-CSF (21).

Based on the data from GM-CSF-treated murine JAGN1-deficient neutrophils (13), we hypothesized that GM-CSF treatment in patients with JAGN1-mutant SCN and insufficient G-CSF response would have a positive effect. However, GM-CSF therapy was unable to rescue the quantitative defect in our two unrelated patients with JAGN1 mutations. Given their severe infectious phenotype hematopoietic stem cell transplantation (HSCT) was ultimately performed to restore the patients’ phagocyte immunity.

## Case description

### Patient 1

**Figure 1:**
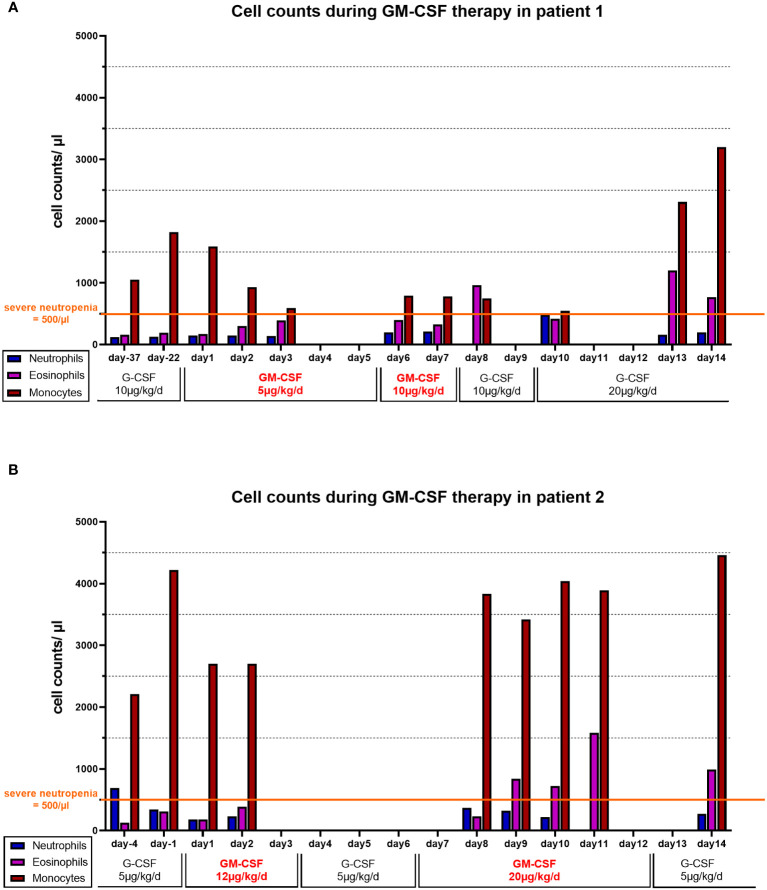
Cell counts during GM-CSF therapy: Absolute numbers of neutrophils (blue bars), eosinophils (purple bars) and monocytes (red bars) at available time points in **(A)** patient 1 and **(B)** patient 2. G-CSF or GM-CSF were administered daily. In Patient 2 **(B)** A three-day break of GM-CSF treatment was required due to organizational matters and was bridged by G-CSF (5 µg/kg/d s.c.).

**Figure 2:**
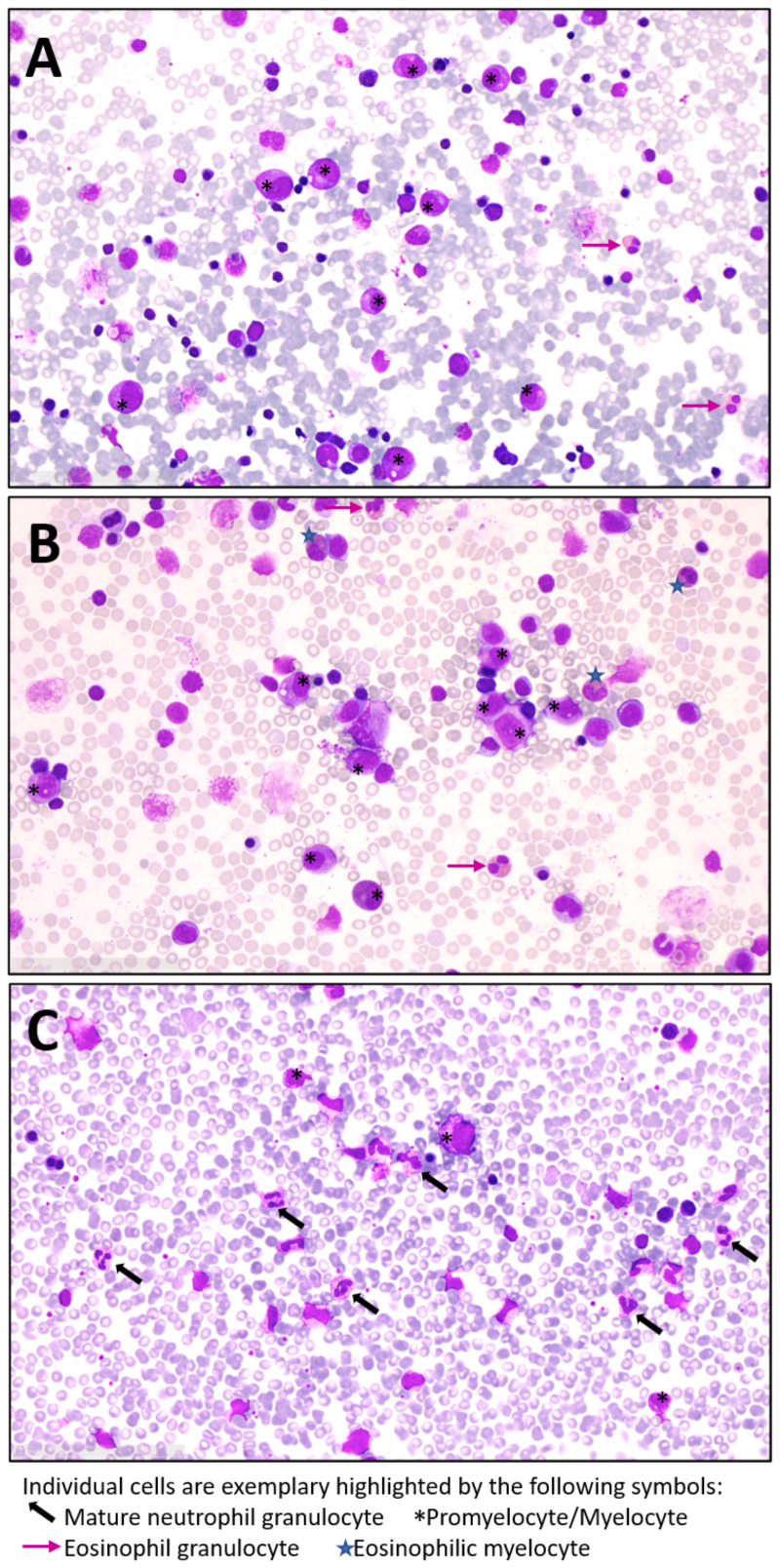
Lack of mature neutrophil granulocytes in bone marrow of our JAGN1-mutant SCN patients: Exemplary microscopic images of bone marrow from **(A)** patient 1, **(B)** patient 2 and **(C)** a healthy control by light microscopy (40x magnification). Bone marrow aspiration samples were spread on slides and samples were stained with May-Grünwald-Giemsa. At the time of bone marrow aspiration, patient 1 was under G-CSF treatment while patient 2 had not yet started with G-CSF.

The patient presented to our clinic at the age of 17 years, diagnosed with SCN of unknown cause during early childhood. Previous infections included a gangrene-induced sepsis with need for intensive care treatment. The patient had no dysmorphisms, no congenital skeletal defects, and did not display symptoms of organ failure. On first admission to our clinic, the patient was on G-CSF treatment with subcutaneous (s.c.) filgrastim at 50 µg/kg/d. Yet, very low neutrophil numbers were noted (around 200/µl). Genetic assessment at that time point only revealed a heterozygous somatic stop-mutation in the intra-cytoplasmatic region of the G-CSF-receptor gene (p.Q741*). At the age of 18 years, the patient became pregnant. After in-depth considerations of potential risks for the fetus, the patient was offered s.c. treatment with GM-CSF (sargramostim/Leukine^®^). Given the previous long complicated course of disease without causal treatment options she agreed with the experimental application after thorough consideration in light of her ongoing pregnancy. At 12 weeks of gestation, GM-CSF was administered for 7 days, initially at 5 µg/kg/d (=165µg/m²/d) for 5 days. As no rise in neutrophils was detected, the dose was escalated to 10 µg/kg/d (=330µg/m²/d). At day 8, the neutrophils dropped below 100/µl and the patient presented clinically with gingivitis, ultimately requiring intravenous (i.v.) antibiotics. In the absence of a response, GM-CSF was stopped and G-CSF was restarted (**Figure 1A**). In spite of G-CSF therapy, the patient´s neutropenia persisted and she was admitted to the hospital repeatedly during her pregnancy with infectious complications such as severe gingivitis, fever of unknown origin, dyspnea, and two episodes of urosepsis. At 40 + 5 weeks of pregnancy, a healthy baby with normal blood cell and neutrophil counts was born. In spite of being on G-CSF, the patient continued to have infectious complications. Bone marrow assessment showed maturation arrest even under G-CSF treatment (**Figure 2A**). In the meantime, genetic testing revealed a homozygous missense mutation in JAGN1 (c.63G>T, p.Glu21Asp), which has previously been reported (4). At the age of 20 years, she underwent HSCT from a matched related sibling. At the last available follow-up two years after HSCT, she had full donor chimerism, normal neutrophil counts and no clinical issues (**Table 1**).

**Table 1:** Demographic features and clinical phenotype.

Patient	1	2
**Sex**	Female	Female
**Mutation (JAGN1)**	c.63G>T, p.Glu21Asp homozygous, The patient´s parents each carried the c.63G>T variant in heterozygous state.	c.36del p.Asp13Thrfs*17 (Exon 1) and c.63G>T, p.Glu21Asp (Exon 1) both heterozygous The mother also carried the heterozygous c.36del p.Asp13Thrfs*17 but not the c.63G>T variant. The father was unavailable for genetic testing. Analysis was performed by Sanger sequencing.
**Mutation (additional)**	Acquired heterozygous stop-mutation in the intra-cytoplasmatic region of the G-CSF-receptor gene (p.Q741*, NP.000751.1, allele frequency 5.7%) No other leukemia associated gene variants were detected.	Not investigated
**Excluded**	HAX1, ELANE, G6PC3	HAX1, ELANE
**Infection history**	- Recurrent infections in childhood (details unknown) - Gangrene with sepsis (including ICU treatment) on G-CSF - Severe otitis on G-CSF - Twice urosepsis during pregnancy on G-CSF - Cholesteatoma with Mastoiditis on G-CSF - recurrent febrile infections (e.g., pneumonia) on G-CSF	- Suspected bacterial infection at the age 6 weeks - Pneumonia at the age of 2,5 and 4 months - Recurrent febrile episodes while on G-CSF (CRP>100mg/l)
**Bone marrow findings**	Majorly maturation arrested promyelocytic compensation with very low myelo, metamyelo, band and granulocytic maturation signs (**Figure 2A**). No signs of malignancy, no blasts, moderate histiocyte activation.	Cell-rich bone marrow with marked promyelocytosis and almost absent maturation of granulopoiesis (**Figure 2B**). Monocytosis and slight eosinophilia. No blasts, normal erythropoiesis.
**Mean neutrophil count on G-CSF**	330/µl [95% CI of the mean 238-422/µl]	301/µl [95% CI of the mean 237-364/µl]
**Mean monocyte count on G-CSF**	1670/µl (age specific reference 200-900/µl)	3900/µl (age specific reference 150-1200/µl)
**Co-Morbidity**	Leg length difference right<left of 3 cm, contracture and valgus position of right knee following severe gangrenous infection of the right thigh. Otherwise, normal growth. Dental abnormalities (recurrent gingivitis).	None, normal growth
**Morphological changes**	None	Triangular face
**Indication for** **GM-CSF**	- Insufficient G-CSF response and recurrent infections - Pregnancy which prompted trial with GM-CSF	- Severe infection phenotype on G-CSF, waiting for HSCT
**Age at** **GM-CSF treatment**	18 years	5 months
**HSCT**	MSD bone marrow transplantation at the age of 20 years **Conditioning therapy:** Fludarabin, Busulfan, ATG **Engraftment:** Leucocytes d18, Thrombocytes d22 **Complications**: CMV-Reactivation, aGVHD intestine grade 1 **Status on last available assessment** (ca. 2 years after HSCT): no signs of GVHD, no relevant infections, no medication other than Vitamin D supplementation. Normal neutrophil counts (1750/µl) and IgG level (10.4g/l). **Chimerism**: full donor.	9/10 MMUD bone marrow transplantation at age 8 months **Conditioning therapy:** Fludarabin, Treosulfan, ATG, Thiotepa **Engraftment:** Leucocytes d16, Thrombocytes d18 **Complications:** CMV-Reactivation, HHV6 in blood, fever **Status on last available assessment** (2 years after HSCT): No signs of GVHD, no infections, no medication other than Vitamin D supplementation. Normal neutrophil counts (2260/µl) and normal IgG level (7.38 g/l) **Chimerism:** full donor.

### Patient 2

A 4-month-old girl with pneumonia, elevated CRP (282 mg/L), anemia (8 g/dL) and severe neutropenia (200/µL) was referred to our hospital. Infection history was positive for neonatal infection at the age of 6 weeks and pneumonia at the age of 10 weeks, requiring inpatient treatment. In the bone marrow, there was a maturation arrest of granulopoiesis in line with a diagnosis of SCN (**Figure 2B**). The patient was immediately started on G-CSF (5µg/kg/d), which led to monocytosis without persistent effect on neutrophil numbers. The patient received posaconazole treatment due to oral thrush and elevated beta-D-glycan. At 5 months, a single dose IgG was given (IgG level 1.35 g/l) (**Table 2**). Two compound heterozygous mutations in JAGN1 were detected (c.36del p.Asp13Thrfs*17 and c.63G>T, p.Glu21Asp). As the patient was highly symptomatic (recurrent febrile infections with CRP > 100 mg/l) and did not respond to G-CSF therapy, hematopoietic stem cell transplantation (HSCT) was recommended, based on case reports for JAGN1 deficiency. While preparing for HSCT, G-CSF therapy was continued without achieving a significant increase in the neutrophils. We offered daily GM-CSF treatment, which was accepted by the mother after informed consent, as she hoped HSCT may be dispensable. A three-day break was required due to organizational matters and was bridged by G-CSF (5 µg/kg/d s.c.). As an initial dose of 12 µg/kg/d (=212µg/m²/d) GM-CSF s.c. did not show any quantitative effect, the dose was subsequently increased to 20 µg/kg/d (350µg/m²/d) s.c. for another 6 days (**Figure 1B**). Total treatment time was 9 days. GM-CSF was discontinued and G-CSF re-started as the neutrophils fell below 100/µl and signs of infection were noted. At the age of 8 months the patient underwent HSCT from a mismatched unrelated donor (9/10). The patient is well 2 years after HSCT showing normal neutrophil counts and full donor chimerism on last assessment (**Table 1**).

**Table 2:** Immunological findings.

	Patient 1	Patient 2
	**Age 20 years (prior to HSCT)**	**Age 5 months (prior to HSCT)**
**IgG (g/l)**	elevated **20.7 g/l** (ref 7-16 g/l)	low **1.35 g/l** (ref 2.2-6 g/l)
**IgA (g/l)**	normal 3.15 g/l (ref 0.7-4.0 g/l)	normal 0.24 g/l (ref 0.08-0.26g/l)
**IgM (g/l)**	normal 1.43 g/l (ref 0.4-2.3 g/l	almost normal 0.24 g/l (ref 0.26-1.0 g/l)
**Lymphocytes**	1460/µl	**10282/µl**
**T-cells (CD3+)**	1139/µl (ref 680-2000/µl)	5964/µl (ref 2300-6500/µl)
**T helper cells (CD3+/CD4+)**	858/µl (ref 310-1185/µl)	3907/µl (ref 1500-5000/µl)
**Cytotoxic T-cells (CD3+/CD8+)**	721/µl (ref 180-820/µl)	**1851/µl** (ref 500-1600/µl)
**B-cells (CD19+)**	**258/µl** (ref 40-140/µl)	**3187/µl** (ref 601-2699/µl)
**NK-cells (CD3-/CD56+)**	**36/µl** (ref 65-535/µl)	1131/µl (ref 201-1199/µl)
**Naive CD4+ cells (CD4+/CD45RA+)**	307/µl (ref 200-640/µl)	3438/µl (ref 1600-6000/µl)
**Memory CD4+ cells (CD4+/CD45RO+)**	373/µl (ref 145-530/µl)	
**Central memory CD4+ cells (CD4+/CD45RA-/CD27+)**		449/µl (ref 53-2200/µl)
**Effector Memory CD4+ cells (CD4+/CD45 RA-/CD27-)**		**27/µl** (ref 2-21/µl)
**Naive CD8+ cells (CD8+/CD45RA+)**	**252/µl** (ref 290-570/µl)	800/µl (345-1635/µl)
**Memory CD8+ cells (CD8+/CD45RO+)**	80/µl (ref 25-320/µl)	159/µl (14-260/µl)
**Effector memory CD8+ cells (CD8+/CD45RO+/CD27-)**		**396/µl** (0-18/µl)
**Effector CD8+ cells (CD8+/CD45RO-/CD27-)**		**498/µl** (0-50/µl)
**B-cell differentiation**	not available	normal distribution of naive, memory and transitional B-cells
**Neutrophil surface markers**	normal expression of adhesion molecules CD11a/CD18, CD11b/CD18, CD11c/CD18	not available
**Neutrophil burst**	sufficient phagocytosis and radical oxygen generation of the granulocytes	not available

### Diagnostic assessment

Both patients’ neutrophil count did not respond to G-CSF therapy resulting in a markedly increased infection history and long-lasting severe neutropenia under G-CSF. They both displayed persistent (patient 2) or recurrent (patient 1) monocytosis in peripheral blood while on G-CSF treatment (**Table 1**). G-CSF therapy did not greatly affect eosinophil counts with mean values of around 220/µl in both patients. On treatment with GM-CSF, the neutrophil count ultimately dropped down below 100/µl in both patients, and GM-CSF was stopped as the patients subsequently developed signs of infection requiring antibiotic treatment. There was a rise in eosinophils under GM-CSF therapy, confirming general efficacy of GM-CSF. In both cases, monocytes were already elevated on G-CSF therapy, and did not show major variations on GM-CSF therapy (**Figures 1A, B**).

In contrast to the persistent severe neutropenia with a mean around 300/µl in both patients, no major other immunological deficiencies were evident (**Table 2**). Both patients showed B-cells above the upper limit, but the adult had hypergammaglobulinemia while the infant had an IgG below the age-specific value. They both had normal IgA and IgM values and the infant displayed a normal B-cell differentiation. At the time of immunophenotyping the infant patient was recently treated for an infection and the lymphocyte panel showed an expansion of particularly CD8 effector and CD8 effector memory T-cells. CD4 T-cell counts were normal in both patients.

## Discussion

### JAGN1-mutant neutrophils are hyporesponsive to G-CSF

G-CSF ameliorates the phenotype in around 90% of patients with SCN by improving neutrophil survival (15, 19, 22). In contrast, JAGN1 patients have been described as low-responders (4, 6). In line with this, both our patients presented with a severe infectious phenotype and neutrophils below 500/µl in spite of G-CSF treatment. JAGN1-mutant neutrophils show increased sensitivity to apoptotic and necrotic stimuli and defective protein glycosylation as well as reduced granules and aberrant granule exocytosis (4). JAGN1 is located at the ER and mutated protein enhances ER stress and apoptosis. Recent data suggests that the induction of calcium- and calpain-dependent cell death in myeloid cells is a major contributor to the manifestation of JAGN1-mutant SCN as evidenced from experiments using HL-60 cells (5). As G-CSF displays part of its anti-apoptotic features via inhibition of calpain and calcium-influx (23), it, however, still remains unclear why many, albeit not all JAGN1 patients are often non-responsive to G-CSF treatment. One potential explanation may be the presence of deficient N-glycosylation of the G-CSF receptor in patients with JAGN1-associated neutropenia, which has been suggested to contribute to inefficient G-CSF-R mediated signaling and thus G-CSF unresponsiveness (4). Notably, several other genes leading to SCN have also be associated to ER dysfunction. Among those are mutations in G6PC3, which also lead to defects in glycosylation. Upon treatment with G-CSF, neutrophil numbers in patients with G6PC3 defects do, however, usually increase (24). Further research is therefore needed to address to which extent aberrant glycosylation patterns in JAGN1 SCN affect the treatment response to G-CSF and whether different JAGN1 mutations lead to diverse functional consequences on the molecular level.

### GM-CSF does not convey protection against bacterial infections in JAGN1-mutant SCN patients

Experiments by Wirnsberger et al. suggested a beneficial effect of GM-CSF on neutrophil function and Candida defense in a mouse model of hematopoietic JAGN1 deficiency as well as in human JAGN1-mutant bone marrow cells (13). In both our patients, however, there was neither an associated rise in neutrophil numbers nor clinical protection from infection under the otherwise well-tolerated GM-CSF therapy. The administered starting doses in our patients were well within recommended standard doses for various bone marrow stimulating applications of GM-CSF. Escalating the doses further did not lead to a beneficial effect. The appearance of infectious signs, concomitantly with a drop in neutrophils below 100/µl, ultimately resulted in discontinuation of GM-CSF therapy. Notably, G-CSF has also been ineffective in some but not all patients with inherited biallelic CSF3R mutations in the extracellular domain of the G-CSF receptor (25), and beneficial effects by GM-CSF have been reported (17, 26). Among the reported cases, 1 patient with compound heterozygous CSF3R germline mutation responded to GM-CSF (final dose 3 µg/kg twice a week) without adverse events over a 12-year period (17). On this dose, the patient’s neutrophil counts remained over > 1000/µl and no infections occurred. Another patient with homozygous germ line deletion in the CSF3R gene was also effectively treated with GM-CSF, and increased neutrophils from < 500/µl to > 1000/µl were already noticed after 2 injections with 5 µg/kg/d every other day and infections disappeared (27). Zhou et al. reported another case of biallelic CSF3R mutation, who was unresponsive to G-CSF, but maintained neutrophils around 1000/µl and remained infection free on a final dose of 3µg/kg/d once a week (16). This argues that the observation time and dose administrated in our patients should have been sufficient to expect a treatment response. Patients with CSF3R mutations frequently display full myeloid cell maturation in the bone marrow in spite of severe peripheral neutropenia (18). Notably, both our patients did show a characteristic maturation arrest in the bone marrow (**Figures 2A–C**), and this was also the case in most but not all patients originally reported (4). The divergent response to GM-CSF in our patients in contrast to the patients with CSF3R mutations and known reduced G-CSF-Receptor signaling underlines that neutrophil maturation and survival in JAGN1-mutant SCN is affected at different levels.

Differentiation from hematopoietic stem cells to mature neutrophils is driven predominantly by G-CSF and to a lesser extent by GM-CSF in combination with other cytokines (28). In healthy individuals, nicotinamide phosphoribosyltransferase (NAMPT) activation has been suggested to be essential for both GM-CSF and G-CSF triggered granulopoiesis (29), and inhibition of NAMPT decreased both G-CSF and GM-CSF-induced colonies from hematopoietic cells *in vitro* (30). In patients with SCN due to ELANE and HAX1 mutations, insufficient activation of NAMPT by GM-CSF, but marked elevation by G-CSF was observed and was suggested to account for the lacking treatment response to GM-CSF in these patients (30). Further functional studies, ideally using primary cells from JAGN1-mutant patients, are needed to assess whether aberrant NAMPT signaling could potentially play a role in the low response for both GM-CSF and G-CSF in JAGN1-mutant SCN.

### GM-CSF may rescue the antifungal killing capacity of JAGN1-mutant neutrophils

Apart from its antiapoptotic properties, GM-CSF is known to enhance cytotoxic Candida albicans killing (28). JAGN1 was found to be required for MPO expression and Candida killing within NETs (neutrophil extracellular traps), and GM-CSF was able to restore the MPO expression in NETs and upregulated calprotectin expression in a model of HL-60 derived neutrophil-like cells with silenced JAGN1 expression (31). Pre-treatment with GM-CSF also restored fungal killing capacities in bone marrow cells from JAGN1 patients (13). While patients with neutropenia may be prone to fungal infections, the risk of severe affection by bacterial infection is much higher. Thus, the known pro-inflammatory properties of GM-CSF are likely insufficient in patients, where neutrophil counts remain below a certain threshold as observed in our patients. Notably, our adult patient, although severely neutropenic and highly susceptible to bacterial infections, did not suffer from any fungal infections, while the infant achieved good fungal control with posaconazole.

### Variable immunological and extra-hematopoietic findings in patients with JAGN1-mutant SCN

Experimental data from a mouse with JAGN1-deficient B-cells suggests a crucial role of JAGN1 for IgG production and humoral immune response (32). In human JAGN1-mutant SCN patients, hypogammaglobulinemia has been reported in some, but not in all patients (6, 11, 33). Our infant patient also had IgG below the age-specific normal values, while the young woman had elevated IgG-levels prior to HSCT. As the adult patient experienced several severe infections prior to HSCT we hypothesize that the hypergammaglobulinemia was caused by constant immune stimulation. In the infant, the reduced IgG value was not accompanied by aberrancies in B-cell differentiation. Specific antibody responses were not available. Although the reason behind the variable immunological phenotype remains to be determined, assessment of immunoglobulin levels and specific antibody responses in neutropenic patients of unknown origin appears reasonable from a clinical point of view.

JAGN1 is ubiquitously expressed, thus extra-hematopoietic manifestations are possible. Regarding the extended clinical phenotype there appears to be quite some variability and no strict genotype-phenotype association has been observed so far (4, 34). Extra-hematopoietic manifestation mentioned in literature are short stature, pyloric stenosis, scoliosis, convulsions, osteoporosis, pancreatic insufficiency, amelogenesis imperfecta, urogenital malformations, hypothyroidism and mild facial dysmorphism (11). In addition, one JAGN1-mutant SCN patient with bleeding abnormalities and recurrent intracranial hemorrhage has also been reported (33). Both our patients did not show systemic abnormalities and only very mild phenotypic variation such as a triangular face in patient 2 as described by others (6). While lack of apparent extra-hematopoietic manifestation may be a reflection of the young age in patient 2, normal findings in patient 1 in spite of a severe neutropenic and infectious phenotype suggests, that additional factors may play a role in the development of extra-hematopoietic features. The low number of reported patients with currently less than 25 hampers our understanding of potential genotype-phenotype correlations. Notably, many of the reported patients are derived of consanguineous families. Thus, there may be additional genes but also environmental factors likely involved in the systemic features, the degree of severity and the presence of additional immunological aberrations.

## Conclusion

GM-CSF did not add any benefit to the clinical care of two patients with SCN due to JAGN1 mutations. Extra-hematopoietic manifestations had no correlation with the severity of the neutrophil and infectious phenotype. More investigations are required to understand the contribution of mutated JAGN1 for the immunological phenotype and the precise mechanism behind the reduced G-CSF responsiveness in most of the reported patients. Given the elevated risk of severe bacterial infections and an associated enhanced malignancy risk in patients requiring high doses of G-CSF (21), JAGN1 patients with documented hyporesponsiveness to G-CSF and severe infection phenotype should be considered early for HSCT.

## Data Availability

The original contributions presented in the study are included in the article/supplementary material. Further inquiries can be directed to the corresponding author.

## References

[1] McDermott DH Malech HL . JAGN1 mutations in severe congenital neutropenia. Br J Haematol. (2021) 192:9–10. doi: 10.1111/bjh.17135 33207009

[2] Skokowa J Dale DC Touw IP Zeidler C Welte K . Severe congenital neutropenias. Nat Rev Dis Primers. (2017) 3:17032. doi: 10.1038/nrdp.2017.32 28593997 PMC5821468

[3] Boztug K Klein C . Genetic etiologies of severe congenital neutropenia. Curr Opin Pediatr. (2011) 23:21–6. doi: 10.1097/MOP.0b013e32834262f8 21206270

[4] Boztug K Jarvinen PM Salzer E Racek T Monch S Garncarz W . JAGN1 deficiency causes aberrant myeloid cell homeostasis and congenital neutropenia. Nat Genet. (2014) 46:1021–7. doi: 10.1038/ng.3069 PMC4829076 25129144

[5] Khandagale A Holmlund T Entesarian M Nilsson D Kalwak K Klaudel-Dreszler M . Severe congenital neutropenia-associated JAGN1 mutations unleash a calpain-dependent cell death programme in myeloid cells. Br J Haematol. (2021) 192:200–11. doi: 10.1111/bjh.17137 PMC7839451 33206996

[6] Cipe FE Aydogmus C Baskin K Keskindemirci G Garncarz W Boztug K . A rare case of syndromic severe congenital neutropenia: JAGN1 mutation. Turk J Pediatr. (2020) 62:326–31. doi: 10.24953/turkjped.2020.02.022 32419428

[7] Cifaldi C Serafinelli J Petricone D Brigida I Di Cesare S Di Matteo G . Next-generation sequencing reveals A JAGN1 mutation in a syndromic child with intermittent neutropenia. J Pediatr Hematol Oncol. (2019) 41:e266–e9. doi: 10.1097/MPH.0000000000001256 30044346

[8] Al-Herz W Chou J Delmonte OM Massaad MJ Bainter W Castagnoli R . Comprehensive genetic results for primary immunodeficiency disorders in a highly consanguineous population. Front Immunol. (2018) 9:3146. doi: 10.3389/fimmu.2018.03146 30697212 PMC6340972

[9] Arunachalam AK Suresh H Edison ES Korula A Aboobacker FN George B . Screening of genetic variants in ELANE mutation negative congenital neutropenia by next generation sequencing. J Clin Pathol. (2020) 73:322–7. doi: 10.1136/jclinpath-2019-206306 31732620

[10] Yilmaz Karapinar D Patiroglu T Metin A Caliskan U Celkan T Yilmaz B . Homozygous c.130-131 ins A (pW44X) mutation in the HAX1 gene as the most common cause of congenital neutropenia in Turkey: Report from the Turkish Severe Congenital Neutropenia Registry. Pediatr Blood Cancer. (2019) 66:e27923. doi: 10.1002/pbc.27923 31321910

[11] Baris S Karakoc-Aydiner E Ozen A Delil K Kiykim A Ogulur I . JAGN1 deficient severe congenital neutropenia: two cases from the same family. J Clin Immunol. (2015) 35:339–43. doi: 10.1007/s10875-015-0156-2 25851723

[12] Welte K Zeidler C Reiter A Muller W Odenwald E Souza L . Differential effects of granulocyte-macrophage colony-stimulating factor and granulocyte colony-stimulating factor in children with severe congenital neutropenia. Blood. (1990) 75:1056–63. doi: 10.1182/blood.V75.5.1056.1056 1689595

[13] Wirnsberger G Zwolanek F Stadlmann J Tortola L Liu SW Perlot T . Jagunal homolog 1 is a critical regulator of neutrophil function in fungal host defense. Nat Genet. (2014) 46:1028–33. doi: 10.1038/ng.3070 PMC6245568 25129145

[14] Ganser A Ottmann OG Erdmann H Schulz G Hoelzer D . The effect of recombinant human granulocyte-macrophage colony-stimulating factor on neutropenia and related morbidity in chronic severe neutropenia. Ann Intern Med. (1989) 111:887–92. doi: 10.7326/0003-4819-111-11-887 2683920

[15] Dale DC Bonilla MA Davis MW Nakanishi AM Hammond WP Kurtzberg J . A randomized controlled phase III trial of recombinant human granulocyte colony-stimulating factor (filgrastim) for treatment of severe chronic neutropenia. Blood. (1993) 81:2496–502. doi: 10.1182/blood.V81.10.2496.2496 PMC4120868 8490166

[16] Zhou J Sun C Huang H Zhu Q Wen F Dong Y . Efficacy of low-dose rhGM-CSF treatment in a patient with severe congenital neutropenia due to CSF3R deficiency: case report of a novel biallelic CSF3R mutation and literature review. Front Pediatr. (2021) 9:746159. doi: 10.3389/fped.2021.746159 34778134 PMC8585998

[17] Klimiankou M Klimenkova O Uenalan M Zeidler A Mellor-Heineke S Kandabarau S . GM-CSF stimulates granulopoiesis in a congenital neutropenia patient with loss-of-function biallelic heterozygous CSF3R mutations. Blood. (2015) 126:1865–7. doi: 10.1182/blood-2015-07-661264 26324699

[18] Triot A Jarvinen PM Arostegui JI Murugan D Kohistani N Dapena Diaz JL . Inherited biallelic CSF3R mutations in severe congenital neutropenia. Blood. (2014) 123:3811–7. doi: 10.1182/blood-2013-11-535419 PMC4055927 24753537

[19] Connelly JA Choi SW Levine JE . Hematopoietic stem cell transplantation for severe congenital neutropenia. Curr Opin Hematol. (2012) 19:44–51. doi: 10.1097/MOH.0b013e32834da96e 22080845 PMC3291495

[20] Germeshausen M Skokowa J Ballmaier M Zeidler C Welte K . G-CSF receptor mutations in patients with congenital neutropenia. Curr Opin Hematol. (2008) 15:332–7. doi: 10.1097/MOH.0b013e328303b9f6 18536571

[21] Rosenberg PS Zeidler C Bolyard AA Alter BP Bonilla MA Boxer LA . Stable long-term risk of leukaemia in patients with severe congenital neutropenia maintained on G-CSF therapy. Br J Haematol. (2010) 150:196–9. doi: 10.1111/j.1365-2141.2010.08216.x PMC2906693 20456363

[22] Ward AC Dale DC . Genetic and molecular diagnosis of severe congenital neutropenia. Curr Opin Hematol. (2009) 16:9–13. doi: 10.1097/MOH.0b013e32831952de 19057199 PMC2720320

[23] van Raam BJ Drewniak A Groenewold V van den Berg TK Kuijpers TW . Granulocyte colony-stimulating factor delays neutrophil apoptosis by inhibition of calpains upstream of caspase-3. Blood. (2008) 112:2046–54. doi: 10.1182/blood-2008-04-149575 PMC2518906 18524991

[24] Desplantes C Fremond ML Beaupain B Harousseau JL Buzyn A Pellier I . Clinical spectrum and long-term follow-up of 14 cases with G6PC3 mutations from the French Severe Congenital Neutropenia Registry. Orphanet J Rare Dis. (2014) 9:183. doi: 10.1186/s13023-014-0183-8 25491320 PMC4279596

[25] Yilmaz Karapinar D Akinci B Sahin Yasar A Hekimci Ozdemir H Onder Sivis Z Onay H . Congenital neutropenia patient with hypomorphic biallelic CSF3R mutation responding to GCSF. J Pediatr Hematol Oncol. (2019) 41:e190–e2. doi: 10.1097/MPH.0000000000001258 30028820

[26] Klimiankou M Mellor-Heineke S Zeidler C Welte K Skokowa J . Role of CSF3R mutations in the pathomechanism of congenital neutropenia and secondary acute myeloid leukemia. Ann N Y Acad Sci. (2016) 1370:119–25. doi: 10.1111/nyas.13097 27270496

[27] Yilmaz Karapinar D Ozdemir HH Akinci B Yasar AS Sivis ZO Onay H . Management of a patient with congenital biallelic CSF3R mutation with GM-CSF. J Pediatr Hematol Oncol. (2020) 42:e164–e6. doi: 10.1097/MPH.0000000000001359 30499904

[28] Mehta HM Malandra M Corey SJ . G-CSF and GM-CSF in neutropenia. J Immunol. (2015) 195:1341–9. doi: 10.4049/jimmunol.1500861 PMC4741374 26254266

[29] Skokowa J Lan D Thakur BK Wang F Gupta K Cario G . NAMPT is essential for the G-CSF-induced myeloid differentiation via a NAD(+)-sirtuin-1-dependent pathway. Nat Med. (2009) 15:151–8. doi: 10.1038/nm.1913 19182797

[30] Koch C Samareh B Morishima T Mir P Kanz L Zeidler C . GM-CSF treatment is not effective in congenital neutropenia patients due to its inability to activate NAMPT signaling. Ann Hematol. (2017) 96:345–53. doi: 10.1007/s00277-016-2894-5 27966038

[31] Khandagale A Lazzaretto B Carlsson G Sundin M Shafeeq S Romling U . JAGN1 is required for fungal killing in neutrophil extracellular traps: Implications for severe congenital neutropenia. J Leukoc Biol. (2018) 104:1199–213. doi: 10.1002/JLB.4A0118-030RR 30106500

[32] Hagelkruys A Wirnsberger G Stadlmann J Wohner M Horrer M Vilagos B . A crucial role for Jagunal homolog 1 in humoral immunity and antibody glycosylation in mice and humans. J Exp Med. (2021) 218(1):e20200559. doi: 10.1084/jem.20200559 32930709 PMC7953624

[33] Thomas S Guenther G Rowe JH Platt CD Shimamura A Levy O . Severe congenital neutropenia due to jagunal homolog 1 (JAGN1) mutation: a case report and literature review. Front Pediatr. (2023) 11:1223191. doi: 10.3389/fped.2023.1223191 37528877 PMC10389042

[34] Hojabri M Farsi Y Jamee M Abolhassani H Khani HHK Karimi A . JAGN1 mutation with distinct clinical features; two case reports and literature review. BMC Pediatr. (2023) 23:206. doi: 10.1186/s12887-023-04024-y 37120535 PMC10148515

